# Empowering Physical Therapists to Address Their Patient’s Inadequate Physical Activity

**DOI:** 10.1093/geroni/igaf122.2112

**Published:** 2025-12-31

**Authors:** Mariana Wingood, Jaime Hughes, Kritchevsky Stephen, Jennifer Brach, Moore Justin

**Affiliations:** Wake Forest University School of Medicine, Winston-Salem, North Carolina, United States; Wake Forest University School of Medicine, Winston-Salem, North Carolina, United States; Wake Forest University School of Medicine, Winston-Salem, North Carolina, United States; University of Pittsburgh, Pittsburgh, Pennsylvania, United States; Wake Forest University School of Medicine, Winston-Salem, North Carolina, United States

## Abstract

One in ten older adults are inadequately active, doubling their falls and frailty risk. Despite this, 65% of physical therapists (PTs) do not assess or address patients’ physical activity (PA). We are addressing this gap by implementing the Physical Activity Vital Sign (PAVS) and Brief Action Planning (BAP), an evidence-based behavior change technique. Our work is guided by the Exploration, Preparation, Implementation, and Sustainment (EPIS) Framework. In the exploration phase, 51 PTs completed a needs assessment that examined potential implementation determinants, including: 1) individual capability and opportunity; 2) PAVS and BAP’s characteristics; 3) clinic culture and infrastructure; and 4) health system’s attitudes and partnerships. During the preparation phase, an implementation team consisting of PTs, clinic supervisor, older adults, and principal investigator co-developed an implementation plan that aims to address three barriers identified in the needs analysis and prioritized based on feasibility and importance. These barriers are lack of knowledge, confidence, and documentation infrastructure. The plan includes training, audit and feedback, site champions, facilitation, and electronic health record-based implementation strategies. We are evaluating the plan in an outpatient physical therapy clinic, where 20 PTs are participating in a year-long implementation pilot. Using a pre-post knowledge assessment and a confidence rating, we identified that our training increased PTs’ knowledge and confidence in assessing and addressing inadequate PA (p < 0.001). Three months into the project, 50% of PTs used the PAVS and 25% completed BAP with >25% of older adult patients. Currently, the implementation team is refining strategies to further increase the adoption rates.

